# Preparation and Evaluation of Virgin Olive Oil Oleogels Including Thyme and Cumin Spices with Sunflower Wax

**DOI:** 10.3390/gels7030095

**Published:** 2021-07-15

**Authors:** Emin Yilmaz, Şahin Demirci

**Affiliations:** Department of Food Engineering, Faculty of Engineering, Çanakkale Onsekiz Mart University, 17020 Çanakkale, Turkey; sahin.demirci@gmail.com

**Keywords:** virgin olive oil, thyme, cumin, sunflower wax, oleogel, sensory

## Abstract

This study aimed to prepare and evaluate virgin olive oil (VOO) oleogels enriched with thyme and cumin spices with sunflower wax (SW) organogelator. Common physico-chemical, structural, thermal, and rheological analyses were completed. Furthermore, aromatic volatiles composition, sensory descriptive analysis, and consumer tests were provided. Results indicated that spice addition does not interfere with gel formation, stability, and gelation time. The oleogels’ color values were affected by the color of the VOO and the spices. The free fatty acidity and peroxide values were within the acceptable limits for virgin olive oils. There were β’ crystal polymorphs, and melting peak temperatures were around 62 °C. Rheological analyses proved that the oleogels were fairly stable under moderate frequencies, maintained their gelled state until around 52 °C, and recovered their shear induced structural loss after force cessation. There were 22 aromatic volatiles quantified in the samples, which originated from the VOO and spices used as ingredients. A trained panel defined the samples using 13 sensory descriptors. Consumer tests proved that the new oleogels were liked by consumers. Overall, this study provided information and the possibility of spice-enriched and spreadable VOO oleogels to enhance per capita consumption of olive oils with new consumption habits.

## 1. Introduction

Olive tree (*Olea europaea* L.) was one of the most important trees cultivated around 6000 years ago in the Mediterranean region, and typically distributed in Mediterranean countries. Spain, Italy, Greece, Turkey, Morocco, and Syria are the main producers, and the European Union (EU) is responsible for around 70% of world olive production. Around 119 registered varieties of olive tree are cultivated in the coastal zones of Turkey. Of these, 30 are the most common and provide around 2 million tons of olives, 190,000 tons of olive oil, and 410,000 tons of table olives. Turkey exports around 55,000 tons of olive oil and 70,000 tons of table olives yearly, according to published 2016 statistics [1,2,3,4].

Olive oil is produced from olive fruits by physical means through stepwise operations of washing, crushing, malaxation (mixing), phase separation (through pressing, centrifugation or percolation), filtering, filling, and storage. Since oil production was achieved only with physical processes and no applied refining, the olive oil is usually called virgin or natural type oil. Depending on the level of free fatty acidity, the oils were classified, and sale prices were usually determined by its quality classes. Generally, olive oil contains around 55–85% oleic acid, followed by 5–20% polyunsaturated, and 8–15% saturated fatty acids. Furthermore, it is a rich source for minor components like phenolics, triterpenes, tocopherols, pigments, phytosterols, hydrocarbons, aromatic volatiles, and others [1,2].

There are many studies about bio-active constituents and health benefits of olive oils, no need to list them here, but some of these studies could be observed from the recent review articles of Jimenez-Lopez et al. [2] and Al-Asmari et al. [3], and even the old reference book of Boskou [1]. Positive health effects of olive oil were linked to its nature (being virgin) and to its main (balance of oleic, linoleic, linolenic acids) and minor components (oleuropein, other olive phenolics, squalene, phytosterols, tocopherols, carotenoids and chlorophyll, and aromatics). Positive health effects of virgin olive oils were listed as: modulation of lipid metabolism, prevention of platelet aggregation, antitumor activities, regulation of blood pressure, prevention and treatment of diabetes, lowering immunological parameters, prevention of osteoporosis, reducing the risk of neurodegenerative diseases, anti-inflammatory properties, relaxing digestive systems, prevention of oxidative stress, anti-microbial properties, and acceleration of healing from illnesses [2,3]. In Mediterranean countries, and now all over the world, enhancement of virgin olive oil per capita consumption has been suggested by health authorities. Olive oils were consumed as liquid cooking oil, as a salad oil and salad dressings, and in margarine formulations. Ground spices added virgin olive oils were enjoyed as breakfast dips in the Mediterranean region.

It has been a goal to provide virgin olive oils in different forms and with diverse flavors to consumers. Consequently, spreadable olive oil oleogels were prepared with carnauba wax and monoglyceride, and evaluated [5], virgin olive oil (VOO)-sunflower and beeswax oleogels were prepared and evaluated against breakfast margarine [6], VOO-beeswax and sunflower wax oleogels were prepared and evaluated by sensory analyses and consumer tests [7], olive oil-propolis wax oleogels were prepared and effects of ultrasound were studied [8], and olive oil-wax esters derived from soybean fatty acid distillate oleogels were developed [9]. Of course, there are many other studies conducted with various organogelators to create VOO and other oleogels [10,11,12,13]. In almost all of these studies, the effects of different organogelators, gelator addition levels, and process conditions were studied relating to common oleogel properties and stability. Only a few studies dealt with sensory analyses, volatiles composition, and consumer tests [7,12,13,14]. VOO was enriched with vitamins E, D, and β-carotene, and 0.5 wt% of overall weight strawberry, banana, and butter aromas were added before creating oleogels with 5 wt% sunflower wax and beeswax [14]. In this detailed study, sensory evaluations, as well as aromatic-volatiles compositions of the oleogels were analyzed. To the best of our knowledge, there is no study reporting spice-enriched oil oleogels.

The objectives of this study were to prepare and evaluate the VOO-sunflower wax oleogels including added thyme and cumin spices. At 10 wt% wax addition and overall 1.0 wt% addition levels of the spices, the VOO oleogels were prepared and analyzed for common physico-chemical, structural, thermal, and rheological properties. Most importantly, descriptive sensory analysis and consumer tests of the prepared oleogels were provided together with aromatics volatile compound analysis. The main aim was to develop new spreadable VOO products to provide new consumption habits (directly spreadable over bread or cracker loaf) to consumers to extend VOO per capita consumption.

## 2. Results and Discussion

### 2.1. Physico-Chemical Properties

The measured physico-chemical properties of the oleogels are presented in Table 1. The gelation time (GT) is the total time elapsed to solidify a melted oleogel under specified conditions. It was also acknowledged that GT depends on the organogelator type and concentration, oil type, melting and cooling conditions, presence of shear and ultrasound, and others [10,12]. Consequently, comparison of different studies seems meaningless, but GT within a study provides an insight into how fast oleogels could be formed. In this study, thyme containing oleogel (SWO-TE) was gelled at 0.75 min and cumin containing oleogel (SWO-CN) was gelled within 1.00 min at ambient temperatures. This was mostly due to the sunflower wax, which yielded gels in similar time scales in previous studies [5,6,7,13,14,15]. It seems that the presence of solid spice particles does not interfere with GT values. A similar finding was also observed for the oil binding capacity (OBC) values. Clearly, sunflower wax (SW) yielded a reasonably strong and able network to entrap the liquid oil, similar to the previous studies listed above. The oleogel samples were also stable at the applied centrifugal force, which indicates a strong gel nature of the SW at 10 wt% with spice present.

The solid fat index (SFI) values of the oleogel samples were 4.33% and 4.30% for the SWO-TE and SWO-CN samples. The values indicate the level of total solids at 20 °C measured by the pulsed NMR instrument. In oleogels, solid content is usually originated from the added organogelator and from the crystallized triglycerides of the oil used. In this study, 10 wt% SW and VOO yielded SFI values at 20 °C, and these values were quite similar to previous studies listed above, which utilized around 2–10 wt% of SW and oils of sunflower seed, hazelnut, and olive. Most table margarines contain around 7–20% SFI at around 20–25 °C, as indicated [16]. Oleogels are semi-solid to solid preparations at serving temperatures with quite low SFI values, proving their health advantage, which is due to the low levels of saturated and trans-fatty acids, respectively.

The color of an oleogel is the sum result of the ingredients used. In this study, the greenish VOO and spices with yellow and red tones were used with cream-toned SW (observe graphical abstract). The VOO used had 58 L value, −11 a* value and 22 b* value as indicated in the materials section. The SWO-CN sample was brighter (66.35 L value) than the SWO-TE sample having 50.88 L value. Since thyme was a darker spice than cumin, this finding was expected. Likewise, cumin-including oleogel had more redness (7.03 a* value) than the thyme-including one (5.38 a* value). Furthermore, yellowness of the SWO-CN sample (31.27 b* value) was significantly higher than that of the SWO-TE sample (22.82 b* value). Furthermore, it was indicated in previous studies that color of olive oil oleogels were quite different than the starting liquid olive oil due to phase change, respectively [5,6,13]. Overall, colors of the samples were observed as greenish-yellow tones, and these colors could be acceptable for olive oil consumers who are accustomed to consuming green-yellow colored VOOs.

To determine the stability, the free fatty acidity (FFA) and peroxide values (PV) of the samples were measured. The VOO used had 0.97% oleate free fatty acidity and 11.5 meq O_2_/kg oil peroxide value. Both FFA and PV values of the SWO-TE sample were significantly higher than those of the SWO-CN sample (Table 2). The Turkish Codex for olive oil permits maximum values of 0.8% oleate as FFA and 20 meq O_2_/kg oil PV for the ‘extra virgin-EVOO’ class, and a maximum of 2.0% oleate as FFA and 20 meq O_2_/kg oil PV for ‘virgin-VOO’ class oils [17]. Similar values were also indicated for EU Council Regulation (EC) No 1234/2007 [18]. Hence, these new spreadable oleogel products could be accepted as ‘virgin class’ olive oil products. Of course, these samples are no longer liquid oils, but spreadable samples with added spices, and could be acceptable by regulations.

### 2.2. Structural Properties

**Figure 1:**
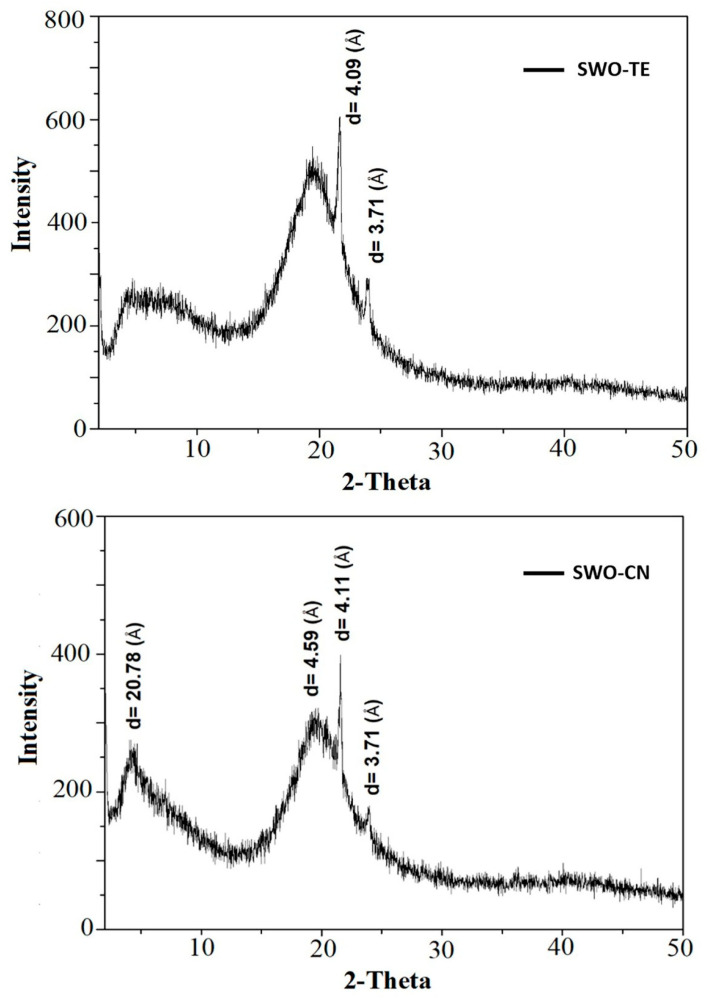
The X-ray diffraction patterns of the oleogel samples (SWO-TE: sunflower wax oleogel with thyme, SWO-CN: sunflower wax oleogel with cumin).

The X-ray diffraction pattern graphics of the samples are shown in Figure 1. Both samples had quite similar patterns. The wide-angle (WAXS) region diffraction peaks of the samples were observed at around 3.71 and 4.09 Å for the SWO-TE sample, and at around 3.71, 4.11, and 4.59 Å for the SWO-CN sample, respectively. According to the official method Cj 2-95 [19], a single peak at d = 4.15 Å indicates the α polymorphic form, two peaks at positions d = 3.8 Å and d = 4.2 Å indicate the β′ polymorphic form, and a peak at position d = 4.6 Å indicates the β polymorphic form.

Consequently, both samples include β′ polymorphic crystal forms. The 4.59 Å peak in the SWO-CN sample does not seem very definite, and might be due to polymorphic transformation. Since both oleogels were prepared from the same VOO and SW at the same concentrations, and the only difference was the kind of the spice added, the presence of the same β′ polymorphic form is quite expected. It has been well documented that polymorphic types of solid fat determine its crystal size, shape, and stabilities. Accordingly, among the four main types of fat polymorphs, γ polymorph (transition state) occurs upon rapid cooling of melted fat. It is transparent, quite unstable, and immediately converts to a more stable α polymorph. The α polymorph is usually characterized with fine, waxy, less stable, least dense, and lowest melting point crystals. Under different cooling regions, β′ type polymorph could form, which yields intermediate density, medium melting point, orthorhombic chain packing crystals with very fine, creamy, soft texture, and immobilizes the maximum amounts of liquid oil due to higher surface area. It was observed that under certain conditions, a fat with β′ polymorph could transform to β polymorph. The β polymorph yields the highest melting point, most dense and stable form, with a coarse and sandy texture. All polymorphs from the same oil result in exactly the same liquid oil upon melting. Generally, polymorph type is based on the oil purity and oil triglyceride configurations, cooling process parameters, storage conditions, shear, and similar factors. In the edible fat industry, certain types of oils were preferred according to their common polymorphic habits to prepare margarine, shortening, and similar products. Sensory quality and palate properties of solid fats were greatly affected by polymorph types, and β′ polymorph was usually preferred in margarine, spreads, and similar products, while β-type polymorph was preferred for sugar confectionery and bakery. It was also acknowledged that different oleogels showed different polymorph types especially according to organogelator type and processing conditions [7,11,12,13,16].

### 2.3. Thermal Properties

Thermal properties of the oleogels were usually measured to determine their similarities to and differences from commercial solid fat products and to determine their melting behavior in mouth space (body temperature). The measured crystallization and melting temperatures and enthalpies are summarized in Table 2.

The SWO-TE sample starts to melt at 48.41 °C and the melting peak occurs at 62.53 °C, while SWO-CN samples’ melting onset and peak temperatures were 53.51 and 62.83 °C, respectively. Clearly, there is no difference for the peak temperatures, but onset (starting to melt) temperatures were different. Thyme spice reduced onset temperature, possibly due to its particle properties causing lipid crystals to melt more easily or possibly due to some compounds leaching from the thyme into the oil. It has been acknowledged that melting temperature of an oleogel is mostly affected by the organogelator type and concentration, oil type (fatty acid composition and triglyceride configuration), and preparation techniques applied [12]. It would be helpful to compare melting data of these sample with literature pertaining to SW oleogels with olive oils. In the study of Yılmaz and Öğütcü [6], olive oil-sunflower wax oleogels were prepared at 3, 7, and 10 wt% SW, and the peak melting temperatures were measured as 58.26, 61.37, and 63.59 °C, respectively. In another study, 5 wt% SW added sunflower seed oil oleogels showed 33.41 °C peak melting temperature [20]. Clearly, both amounts of added wax and oil type caused the differences in the measured melting temperatures. Oleogels prepared in this study had very similar thermal behavior to previously prepared similar VOO-SW oleogels. Hence, added spices have not interfered much with the melting habits. Further, it was indicated that most commercial breakfast and kitchen margarines and spreads were required to quickly melt at slightly above body temperature for cooling sensation on the palate without lingering greasiness or waxiness. Commercial shortenings are said to have somewhat higher melting temperatures based on their usage purpose [16]. Spice-flavored oleogels intended to be used as spreadable fat-like preparations in this study showed somewhat higher melting temperatures, similar to almost all of the previous wax oleogels [6,7,12,14,20]. Therefore, the suitability of these new oleogels as spreadable products and consumer attitudes towards them were evaluated by sensory analysis and consumer tests, and are discussed later.

### 2.4. Rheological Properties

At the beginning of rheological analyses, amplitude sweep tests for the samples were conducted to determine the non-destructive deformation range (the linear viscoelastic region, LVR) and upper limit of this range. Consequently, between 0.01 and 100% strain, 1 Hz frequency at 10 °C, the amplitude sweep tests were conducted and the LVR strains of the samples were determined as 0.014% and 0.015% for the SWO-TE and SWO-CN samples, respectively.

**Figure 2:**
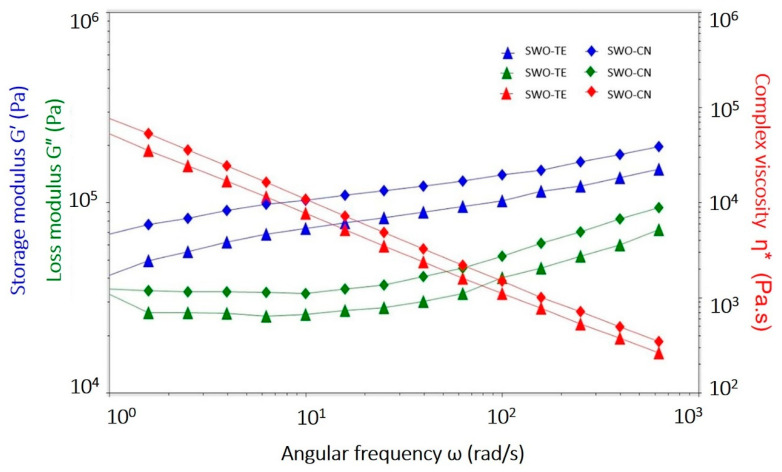
The frequency sweep test graphics of the oleogel samples (SWO-TE: sunflower wax oleogel with thyme, SWO-CN: sunflower wax oleogel with cumin).

Applying the frequency range of 0.1–100 Hz at LVR strains and 10 °C constant temperature, the frequency sweep tests were completed. The frequency sweep graphics are given in Figure 2. Frequency sweep tests usually provide the time-dependent behavior of a sample in the non-destructive deformation (LVR) range. Generally, this test yields information about behavior and inner structure of a gel, as well as its storage stability. High frequencies simulate fast motion on a short time scale, while low frequencies simulate slow motion in long time scales. In this study, we preferred low-to-medium frequencies, which most food products would face during processing and distribution [21]. The changes in the storage (G′) and loss (G″) modulus of the samples can be observed from the graphics. The SWO-TE sample had 13,000–100,000 Pa storage modulus and 12,000–14,000 Pa loss modulus values within the applied frequency range. Similarly, the SWO-CN sample had 16,000–101,000 Pa storage and 12,000–14,000 Pa loss modulus values. In both oleogels, storage modulus values were higher than those of the loss modulus values (G′ > G″), indicating that the materials were mostly similar to solid nature, in other words, they were true-gels.

Within the applied frequency range, the G′ > G″ condition prevails, and this condition proved that the samples were in a gel state. Descriptions of complex viscosity (η*) are useless in practice since for low frequencies, the η* curve approaches infinity. In rheology science, it was stated that the G′ of a sample resembles solid-like properties and describes the elastic portion, while the G″ resembles liquid-like or viscous properties. Consequently, if a sample had higher G′ values than G″ values, it would be more like a solid. In the samples of this study, this means that the prepared oleogels were in real gel-state, and must have enough storage stability since through all applied frequency ranges, the condition was maintained. Furthermore, during the measurement range, no crossover point (G′ = G″) was observed, indicating gel stability. Further, the loss factor (G″/G′ ratio) values ranged between 0.14 and 0.92 for SWO-TE, and between 0.14 and 0.75 for SWO-CN samples. This ratio indicates that these oleogels were also resistant to syneresis. Very similar findings were found previously for the SW and other plant wax oleogels [6,7,10,12,14,20,21].

**Figure 3:**
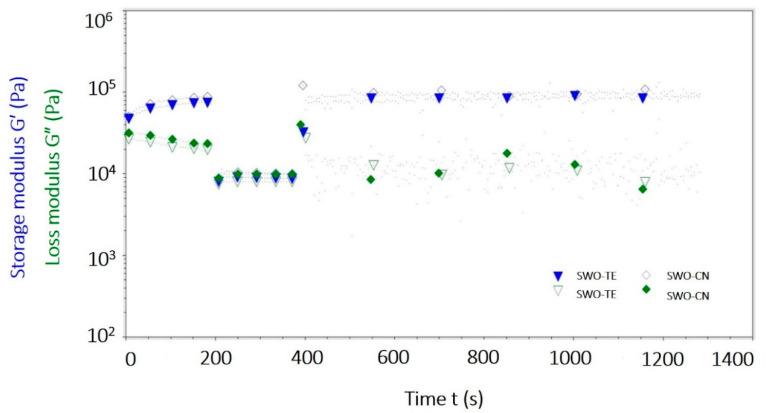
The time sweep test graphics of the oleogel samples (SWO-TE: sunflower wax oleogel with thyme, SWO-CN: sunflower wax oleogel with cumin).

Time-dependent oscillatory viscoelastic behaviour of the prepared oleogel samples were evaluated by time sweep tests (Figure 3). In these types of tests, both amplitude and frequency (1 Hz) were kept constant, and three different shear values were applied at three different time-domains to simulate the resting, destruction, and recovery-regions, as explained in the methods section.

Both oleogel samples were in gel state at resting condition under the LVR strains at the first region, respectively. In the second-domain, high strain was applied purposefully to destroy the structure, and both oleogels were deformed and became free flowing liquids, as evidenced from the lowering G′ and G″ values. Clearly the storage modulus values of both samples were lowered below their loss modulus values (G′ << G″), indicating that the gel network was broken down by mechanical effects. Most importantly, at the third time-domain, the high strain was removed from the samples, and samples re-gelled again, as evidenced from the enhancing G′ values, which again became higher than their loss modulus values to yield the G′ > G″ condition again. Consequently, this test proved that both samples were able to recover their lost structure due to high strain as soon as the strain was lowered. This behavior was typical in most wax oleogels studied before [6,8,9,13]. Overall, the mechanical stability of the prepared oleogels indicated that these new spreadable VOO preparations could be stable during food processing and distribution operations, where mixing, agitation, and some mechanical stress would be unavoidable. If the gel breaks down, then, after the cease of energy input, it can re-gel to provide solid-like structure to thus be spreadable again. This could be accepted as an advantage.

**Figure 4:**
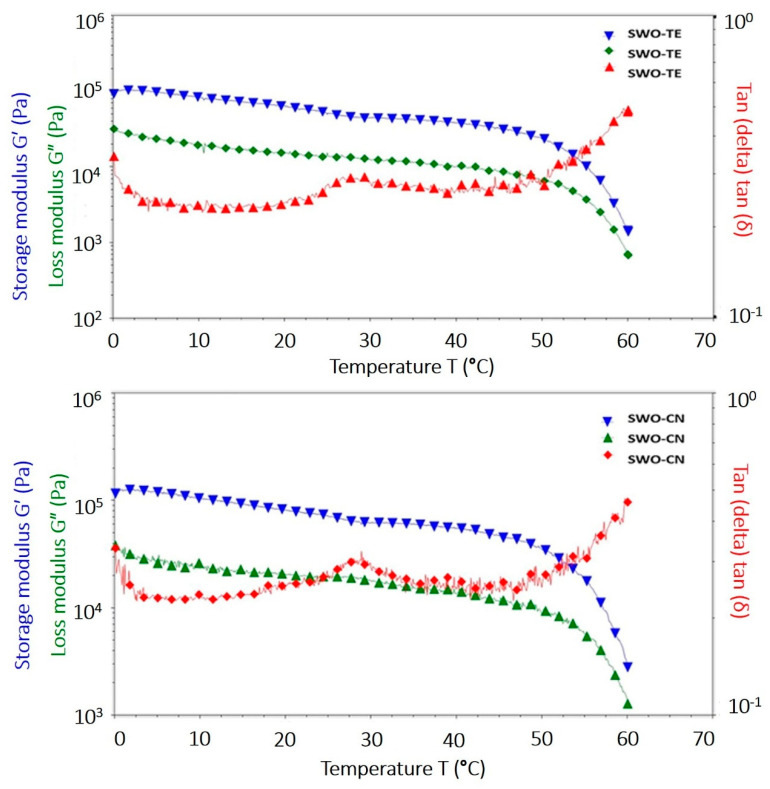
The temperature ramp test graphics of the oleogel samples (SWO-TE: sunflower wax oleogel with thyme, SWO-CN: sunflower wax oleogel with cumin).

The temperature ramp test of the samples was presented in Figure 4. These graphics provide the data to observe changes in the storage and loss moduli during the sample heating process under constant strain and frequencies. Consequently, these changes provide information about how the oleogels respond to temperature enhancements. Clearly, in both samples, the G′ and G″ values were almost constant and linear as temperature increases from 0 to around 52 °C. In this range the samples were well solidified and gelled. Softening of both gels starts at around 52–53 °C, and complete melting occurs at around 60 °C as the crossover point (G′ = G″) is reached. After the crossover point, the oleogels became free flowing liquids. These data concur with DSC-determined melting peak points, which indicate around 62 °C melting temperature. While DSC data provides only melting onset and peak temperatures and enthalpies, these rheological temperature ramp graphics provide the opportunity to follow gel structure during the heating process. Accordingly, these spreadable VOO oleogels would remain solid until around 52 °C, and hence would remain solid in summer season at ambient temperature. This condition has always been mentioned as an advantage of oleogels not requiring refrigeration during handling [12,14,20]. Further, these new oleogels would remain a little longer in mouth space but eventually would melt to yield a good palate.

### 2.5. Volatile Aromatics Composition of the Oleogels

The headspace SPME-collected and GC-MS-quantified volatile aromatics compositions of the prepared oleogel samples are presented in Table 3. The table shows the retention times, aroma descriptions, and mean peak % values of the determined volatiles. There were 22 compounds quantified in the SWO-TE and 20 compounds in the SWO-CN samples. Among them, 12 compounds (ethanol, 1-propen-2-ol-acetate, acetic acid, ethyl acetate, 1-penten-3-ol, hexanal, E-2-hexenal, cymol, limonene, gamma-terpinene, nonanal, farnesene) were common in both samples.

These compounds were usually described with ethereal, fruity, pungent, green, fresh, terpenic, citrus, and woody aroma descriptors. Since both oleogel samples were formulated with the same VOO and SW, most of these common volatiles must have originated from these ingredients. However, some would come from the spices (thyme and cumin), and would be common in both spices as well. Since the volatiles must come from the ingredients used to prepare the oleogels, it would be helpful to compare volatiles of the oleogels with the volatiles of VOO, SW, and the spices published in the literature.

As a virgin and natural oil, olive oils contain more than 100 listed volatile aromatic compounds [1]. A very comprehensive recent review about volatile aromatic compounds, their analysis, and sensory perceptions of various olive oils were published [22]. In this review, a pair of tables listed approximately 700 volatiles identified from olive oil samples in different studies. Most of these volatiles were characterized by molecular weight < 300 Da, with high and variable volatility, variable solubility, and capability to bind proteins or sensory receptors. When literature listed VOO volatiles were compared with the volatiles in Table 3, it was observed that most of the identified volatiles in oleogel samples were listed in VOO samples in the literature. Only acetol, diethyl ketone, geranyl butyrate, cymol, 2-caren-10-al, carvacrol, and bergamotene were absent among the volatiles listed for VOO in the current literature, but were found in the oleogel samples prepared in this study. Consequently, these compounds must come from the spices or SW used. The VOO volatiles identified in the oleogel samples were defined with green, grassy, fruity, musty, fatty, leafy, tomato, and sour aroma terms.

To determine the aromatic volatiles originating from thyme spice in the SWO-TE sample, Table 3 and literature about thyme volatiles were compared [23,24]. The aromatic volatiles of beta-pinene, beta-phellandrene, beta-myrcene, carene, limonene, beta-ocimene, gamma-terpinene, 2-carene, carvacrol, and bergamotene found in the oleogel sample were listed among the volatile constituents of thyme spice in the literature. These volatiles were associated with terpentine, herbal, spicy, minty, pine, woody, and citrus aroma notes.

Similarly, volatiles coming from the added cumin in the SWO-CN oleogel sample were identified by comparing with the literature indicating the volatiles found in cumin samples [25,26]. After screening Table 3 with these references, the volatiles originating from cumin spice in the SWO-CN oleogel were determined as beta-pinene, beta-myrcene, gamma-terpinene, 2-caren-10-al, and carvacrol. These volatiles were identified with herbal, spicy, citrus, and woody aroma descriptions.

Generally, volatile aromatic compositions of the oleogel samples were in agreement with literature regarding the volatiles of the VOO and the spices. The volatile aromatic compositions of the spices alone as solid samples were not measured in this study, since it is well known that aromatic release of spices is strongly dependent on the food matrix, and the chemical nature (polar or non-polar) of the matrix [27]. Consequently, we only referred to literature to identify the volatile aromatics originating from the spices used. Unfortunately, there is no data available regarding the volatile composition of sunflower wax (SW). In fact, the SW used in this study was quite pure, refined, faint, and odorless, as described by the producer. We assume that compounds yielding some fatty, aldehydic, and possibly waxy aroma notes must have originated from the SW. Since the literature about volatile aromatics composition, sensory descriptions, and consumer tests for various oleogels are scarce, these data would contribute significantly to the oleogel literature. The volatile aromatics data could provide information about the kinds and amounts of the chemical compounds responsible for the perceived aroma but cannot provide a human perception of the attributes and whether the consumer would accept the samples or not. Therefore, descriptive sensory analyses and consumer tests were also completed in this study.

### 2.6. Descriptive Sensory Analysis

A trained sensory panel decided to describe the oleogel samples with 13 sensory descriptor terms, and the collected data are summarized in Table 4. Sensory ‘hardness’ was described as the force needed to push a knife into the sample, and the maximum and minimum points (10 and 0 scores) were referenced with tallow fat and yoghurt. Both samples had around 8.15 and 8.25 scores, indicating that the oleogels were not as hard as tallow, but much harder than yoghurt. In fact, they were similar to breakfast margarines. These sensory scores were also in good agreement with the rheological data given in Figure 2. Both oleogel samples were found as fully ‘spreadable’, in comparison with the cream cheese reference for a 10-point score. This finding was observed as quite fulfilling since the goal was to develop spreadable VOO oleogels with added spices. Apparently, spice particles did not impact spreadability. The ‘liquefaction’ was defined as the amount of fat melting during spreading onto the bread surface. It measures the amount of melting by mechanical energy input. Both samples had fairly low (0.50) liquefaction scores. ‘Sandiness’ was defined as the perceived gritty texture on the tongue, and was found as 1.00 for both samples, in comparison with 10 score for semolina as the reference. The fresh olive oil itself was the 10-score reference for the ‘olive fruit’ descriptor, and the oleogels had around a little less than half (4.00–4.20) of the full-score. Since oleogels include SW and most importantly the aromatic spices, this decrease of ‘olive fruit’ description was expected. The ‘grassy’ score of the SWO-CN sample (7.00) was significantly higher than that of the SWO-TE sample (5.15). Usually, fresh VOO was characterized with a grassy or green aroma, and this difference could be attributed to the differences of the aroma potencies of the spices added. Clearly, some grassy notes were masked more by the thyme spice.

The ‘waxy’ scores of both samples were low (0.5) and not different. In previously prepared olive oil-SW oleogels the waxy scores were a little higher than this study [6,14]. The aromatic spices have masked the waxy odor attribute coming from the SW used. In fact, this situation could be well accepted, since the higher waxy odor was not preferred in oleogels [12,13,14,20]. In all fat-containing and long-term stored foods, ‘rancid’ was defined as aromas associated with oxidized oil, and referenced with used frying oil. The oleogels luckily had very low (0.50–0.52) rancid scores. Since fresh VOO was used, and the spices added were strong anti-oxidants, not much oil oxidation occurred during oleogel preparation. ‘Thyme’ and ‘cumin’ were defined for the oleogels with the spices themselves as the references. Of course, each of these aromas was only detected in the corresponding samples. The panel perceived more thyme aroma (5.23) in the SWO-TE sample than cumin aroma (3.00) in the SWO-CN sample (Table 4). Of course, aroma intensity and volatility in oil media differ for the different spices, although both were added at 1 overall wt% level. The ‘hay’ attribute had around 1.00 score in the samples, and it was low but still perceivable. It was associated with dry straw and most probably originated from the added spices. As a mouth feeling attribute, ‘cooling’ was defined as the cold feeling inside the mouth during oleogel consumption. Compared to 10 score of menthol candy, the oleogels had around 2.05 and 2.55 scores. In fact, solid fats and chocolate provide some cooling sensation during mastication in mouth due to heat absorbed by the melting fat crystals [16]. Since oleogels had some crystallized components, this sensation was perceived. ‘Mouth coating’ was defined as the perceived fatty coating on the palate and referenced with butter. The oleogels had a lower mouth coating than butter, but it was quite sensible. Overall, descriptive sensory analysis data is indispensable to provide real human sense descriptions of a food sample to the readers and has been very important in product formulations, comparison, and modifications to end up with successful samples. In fact, results of consumer tests were evaluated with sensory descriptive analysis and volatiles’ data to determine the defects of the sample to take corrective actions or to improve the attributes of the sample to achieve higher consumer acceptance. Of course, the other analytical data (physical, rheological, thermal, etc.) can serve the same purpose.

### 2.7. Consumer Test

It has been stated [7,27] that the bottom line in the market success of any new food is its consumer acceptance. Consequently, a limited consumer test with 50 volunteer participants was conducted (Table 5). The appearance, spreadability, aroma, flavor, and acceptability of the oleogels were tested with 5-point hedonic scale (1 = dislike extremely to 5 = like extremely). There was no significant difference between the samples for the measured properties. The appearance of the oleogels were a little above the ‘liked’ (4.0) score. A similar condition was evident for the spreadability, and spreadability scores were a little higher (4.40 and 4.10). The oleogels had around 4.18 aroma, and 4.36–4.24 flavor scores for the SWO-TE and SWO-CN samples, respectively. Among all attributes measured, the aroma scores were lower, and hence, some improvements for aroma could be suggested. The overall acceptability of the oleogels was above 4.00 score, indicating that the samples were liked by the consumers. In fact, these hedonic data proved that the new oleogels were quite successful in fulfilling consumer expectations. We suggest consumer tests with a much higher number of participants after the worldwide COVID-19 pandemic to expand the results. Furthermore, a consumer test with some product information provided could be conducted to get a wider response from consumers. The consumers could be informed of the nature, formulation, and advantages of these new oleogels before the tests are conducted.

## 3. Conclusions

In this study, VOO-SW oleogels were prepared with added thyme and cumin spices, and evaluated thoroughly. The SW concentration was 10 wt%, and each spice was added at 1% of overall oleogel weight. Physical analyses showed that added spice particles did not interfere with GT, OBC, SFI, and oleogel centrifuge stability. The new oleogels had color values depending on the color of VOO and the spices added. The FFA and PV were within the acceptable limits of the virgin class olive oils. There were β′ polymorph type of crystals in the oleogels. DSC-measured thermal data and rheological temperature ramp tests proved that the prepared oleogels were fairly solid at room temperatures, and quickly melt at just above body temperature at around 52–60 °C. Further, the samples were quite stable under moderate frequencies, and proved to have storage stabilities. The prepared spice including oleogels were both thermo-reversible and mechano-reversible after ceasing high temperatures or high strain was applied. There were 22 and 20 aromatics volatiles quantified in the SWO-TE and SWO-CN samples, and most of these volatiles were shown to originate from the VOO and the spices added. Quantitative sensory descriptive analysis proved that the oleogels would be described by these 13 attributes, and there were not many differences between the samples. Further, most sensory properties were in accordance with other solid and spreadable fat products studied in the literature. Consumer tests indicated that the samples were mostly liked by the consumers and well accepted. This study proved that solid spice particles added to stable VOO oleogels would be prepared successfully to provide alternative spreadable products for olive oil consumers. Since positive nutrition and health effects of olive oils were now accepted all over the world, expansion of per capita olive oil consumption with these types of new spreadable products could be very important. The findings and conclusions of this study could get attention from olive oil and spreadable fat product producers. Similar researches with diverse spices at different addition levels and different organogelators were also anticipated.

## 4. Materials and Methods

### 4.1. Materials

The virgin olive oil (VOO) produced in October 2020 in a two-phase centrifugation system factory located in Çanakkale (Turkey) was purchased, and used for the oleogel preparations. The fatty acid composition of the VOO used was 0.05% myristic acid, 7.2% palmitic acid, 1.3% palmitoleic acid, 3.2% stearic acid, 78.9% oleic acid, 8.2% linoleic acid, 0.5% linolenic acid, 0.5% arachidic acid, and 0.3% behenic acid. Its free fatty acidity was 0.97% oleic acid, and peroxide value was 11.5 meq O_2_/kg oil. The instrumental color values were 58 L value, −11 for a* value, and 22 for b* value. First-grade ground thyme and cumin were bought from a local market. The sunflower seed wax (6607L) was provided by Kahlwax Co. (Kalh GmbH & Co., Trittau, Germany). The ingredients used to prepare the oleogels in this study could be viewed in the graphical abstract provided. All standards, chemicals, and solvents used were of analytical grade and purchased from Sigma Chem. Co. (St. Louis, MO, USA) and Merck (Darmstadt, Germany).

### 4.2. Preparation of the Oleogels

**Figure 5:**
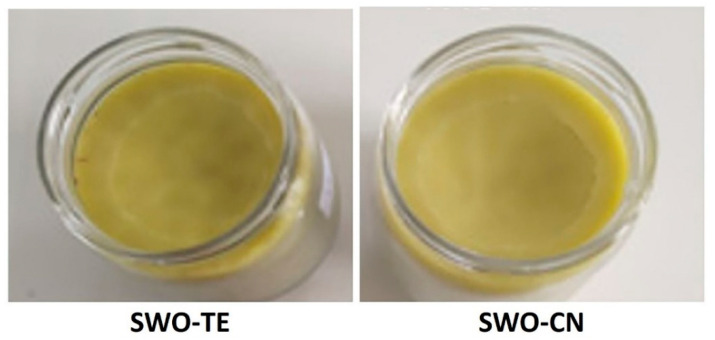
The prepared oleogel samples (SWO-TE: sunflower wax oleogel with thyme, SWO-CN: sunflower wax oleogel with cumin).

The concentration of sunflower wax (SW) in the oleogels was selected as 10 wt%, according to some pre-experiments. The SW used in this study had a 1.0% critical gelling concentration (C*), as we determined in our previous study [13]. Since oleogels in this study included 1.0 wt% overall oleogel spice weight (thyme and cumin) as dry, small particles, we decided on 10 wt% SW to achieve strong enough oleogels as spreadable and stable fat products. Of course, lower levels could be used, but lower levels (8 wt% and lower) were not as hard as spreadable fat products. Consequently, we prepared 500 g of oleogels with 450 g VOO, 50 g SW, and 5 g of each species. First, the weighed amounts of VOO and SW were mixed in a glass beaker and heated at 80 °C for around half-hour in a water bath to fully melt and mix the wax in the oil. The weighed amount of spice was then added, and the hot mixture was homogenized at 3000 rpm for 5 min to disperse the spice particles in the oil. Finally, the mixture was left at ambient temperature controlled with an air-conditioner (20 ± 5 °C) overnight for gelation. The next day, analysis of the prepared oleogel was immediately started. During the analyses, the oleogel samples were kept in the fridge. The prepared oleogels can be observed in Figure 5.

### 4.3. Physico-Chemical Analyses of the Oleogels

The gelation times (GT) of the two kinds of oleogels were measured according to the method explained previously [13,15]. The gelation time at the ambient temperature was provided as minutes.

The oil binding capacity (OBC) was also measured following the previously described technique precisely [13,15].

The centrifuge stability test was conducted by applying 1300× *g* centrifugation force for 15 min at room temperature to the 5 g oleogel sample placed in tubes, and then determining the stability of the gel by visual inspection.

The solid fat index (SFI) of the oleogel samples was measured with a Minispec Bruker NMR Analyzer mq20 (Bruker Optics, Inc.) following the ISO method [14]. The instrument was calibrated with standards including 0, 31, and 73.5% solid fat. The SFI% were measured at 20 °C accordingly [14].

The instrumental color values were measured with a Minolta CR-400 (Konica Minolta Sensing, Osaka, Japan) colorimeter according to CIE standards for the L, a*, and b* values [6].

The free fatty acidity (FFA, oleic%) and peroxide values (PV, meq O_2_/kg oil) of the oleogels were measured with the methods of Ca 5a-40 and Cd 8-53 [28].

### 4.4. X-ray Diffraction Patterns of the Oleogels

An X-ray diffractometer (PANalytical Empyrean model, Almelo, The Netherlands) was used to measure the polymorphic types of the oleogel samples following the Cj 2-95 method [19]. The oleogel samples were kept at ambient temperature overnight and loaded at that temperature to the instrument. The measurement was completed with a Cu source X-ray tube (λ = 1.54056 Å, 40 kV and 40 mA) and angular scans (2θ) from 2.0 to 50° at 2°/min scan rate. X’Pert HighScore Plus software (Malvern Panalytical Ltd., Royston, UK) was used for data analysis [13].

### 4.5. Thermal Analyses of the Oleogels

A Perkin-Elmer 4000 Series Differential Scanning Calorimeter (DSC) (Groningen, The Netherlands) calibrated with Indium and Zinc was used to determine the crystallization and melting onset and peak temperatures, and enthalpies. Around 8–10 mg of each sample was placed into the aluminum pans and sealed. The temperature program was: heat samples from 20 to 100 °C at 10 °C/min rate; cool samples from 100 to −30 °C at 10 °C/min rate, maintain temperature for 3 min for full crystallization, and finally heat samples again to 100 °C at 10 °C/min heating rate. Through this thermal cycling program, both crystallization and melting temperatures and enthalpies were determined simultaneously. Pyris 1 Manager Software was used for the calculations [14].

### 4.6. Rheological Analyses of the Oleogels

All rheological measurements were carried out at 10 °C and under the LVR strains, unless otherwise indicated. A DHR 2 rheometer (TA Instruments, New Castle, DE, USA), donated with cross-hatched parallel plate geometry (*φ* = 40 mm, gap 0.9 ± 0.1 mm), and Peltier system (±0.1 °C) under the lower plate was used. The linear viscoelastic region (LVR) was determined with an amplitude sweep test (0.01–100% strain and 1 Hz frequency) at first for the validity of all further analyses. The LVR is defined as the region where a plateau for the storage (G′) and loss (G″) moduli was observed, which ensures the non-destructive strains for each sample [21].

The LVR strains were 0.014 and 0.015% for samples SWO-TE and SWO-CN, respectively. Frequency sweep tests were completed at 10 °C, with LVR strains and frequencies from 0.1 to 100 Hz. This test provides information about the stability and long-term durability of the gels. The graphics with storage (G′) and loss (G″) modulus values were provided.

The structural recovery abilities of the oleogel samples were evaluated with time sweep tests. This test provides information about samples’ structural recovery abilities when exposed to stress and released from the stress. There were three time domains as time-shear settings to simulate the conditions of resting, structural breakdown, and recovery conditions. The tests were done at 10 °C with 1 Hz frequency. The first domain was applied for 180 s with LVR strains to simulate the resting condition. In the second domain, strains well above LVR strains (1.0% constant) were applied for another 180 s to simulate structural breakdown conditions. Finally, strains much lower than those of the LVR strains (0.001% constant) were applied for 900 s to simulate the structural recovery (resting) domain.

The effects of surrounding temperature change on oleogel structure were tested with a temperature ramp test. The test was achieved by heating the samples from 0 to 80 °C by 1 °C/min heating rate at 1 Hz frequency within the LVR strains.

### 4.7. Volatile Compound Analysis of the Oleogels

The methods of Krist et al. [29] and Yılmaz [30] were followed to determine and quantify the volatile compounds present in the oleogel samples. The analysis was achieved with a Shimadzu GC-2010 Plus gas chromatograph equipped with an MS-QP2010 plus mass spectrometer (Shimadzu Corporation, Kyoto, Japan). The method was an SPME technique, and the volatiles were collected on a fused silica SPME CAR/PDMS (75 mm Fused Silica, Supelco Ltd., Bellefonte, PA, USA) fiber. First, 2.0 g of oleogel sample was weighed into a 15 mL clear PTFE/silicone septa (Supelco) vial, and the vial placed into a water bath set 60 °C. The vial was kept in a water bath for 15 min without fiber, and 30 min with the fused silica SPME fiber. The volatiles adsorbed fiber was then inserted into the injection port of the GC, and the volatiles were desorbed at 250 °C for 5 min. An Rx-5Sil MS capillary column (30 m—0.25 mm × 0.25 mm; catalog no: Restek 13623, Restek, Bellefonte, PA, USA) was used for compound separation. The injection port and the detector were at 250 °C, and helium carrier gas flow rate was 1.61 mL/min. The column was held at 40 °C for 2 min and then increased to 250 °C at a heating rate of 4 °C/min, and then kept for 5 min at that temperature. The temperatures of the ion source and the transfer line were 200 and 250 °C. Electron impact mass spectra were recorded at the ionization energy of 70 eV. The mass spectra analyses were performed in scan mode in the 40–300 amu mass range. Finally, the volatile compounds were tentatively identified by Wiley, Nist, Tutor, and FFNSC mass spectra libraries. Retention times and % area values of the determined volatiles were provided.

### 4.8. Descriptive Sensory Analysis of the Oleogels

The descriptive sensory attributes of the oleogel samples were identified and quantified by a trained sensory panel of 11 panelists (7 female, 4 male, aged 20–50 years) with the quantitative descriptive analysis (QDA) procedures [7,27]. The panel was trained by the panel leader for at least 10 h at different sittings on different days over a week to select, define, and standardize the sensory terms. A consent form was signed and provided to the panel indicating the edibility and safety of the samples. The panel defined 13 descriptive sensory terms, and the attribute definitions and references are given in Table 6. A 10 cm line scale anchored between 0 at the left end for minimum intensity and 10 at the right end for maximum intensity was used for quantification. In each session, the two samples coded with 3-digit numbers were served to the panel. Duplicate samples were served in different sessions randomly. All tests were carried out at room temperature under daylight, and the panelists were provided with water, bread slices, apple slices, and an expectoration cup.

### 4.9. Consumer Tests of the Oleogels

To assess the consumer perception about appearance, spreadability, aroma, flavor, and acceptability of the new oleogels, 50 volunteer consumers participated in the test twice on different days. Coded samples were placed into transparent glasses covered with a lid, and served to the consumers at room temperature together with slices of bread, a plastic knife, a slice of apple, water, and expectoration cups. A 5-point hedonic scale (1 = dislike extremely to 5 = like extremely) was used to collect the data.

### 4.10. Statistical Analysis

The two oleogels were prepared at two different times as two replicates, and each replicate production sample was analyzed in triplicate. The collected data were given as mean values with standard deviations. The analysis of variance (ANOVA) and Tukey’s test, and for the sensory data, the Kruskal–Wallis tests were completed. The level of confidence was at least 95% for all. Statistical analyses were done with Minitab v.16.1 software [31].

## Figures and Tables

**Table 1:** The physico-chemical properties of the prepared thyme and cumin-flavored virgin olive oil-sunflower wax oleogels.

	SWO-TE	SWO-CN
Gelation Time (min)	0.75 ± 0.0 ^b,†^	1.00 ± 0.0 ^a^
Oil Binding Capacity (%)	99.99 ± 0.2 ^a^	99.99 ± 0.0 ^a^
Centrifuge Stability	Stable	Stable
Solid Fat Index (20 °C, %)	4.33 ± 0.5 ^a^	4.30 ± 0.2 ^a^
Color L value	50.88 ± 0.8 ^b^	66.35 ± 0.2 ^a^
Color a* value	5.38 ± 0.1 ^b^	7.03 ± 0.2 ^a^
Color b* value	22.82 ± 0.5 ^b^	31.27 ± 0.4 ^a^
Peroxide Value (meq O_2_/kg)	16.76 ± 1.5 ^a^	12.48 ± 0.7 ^b^
Free Fatty Acidity (oleic %)	2.14 ± 0.1 ^a^	1.95 ± 0.0 ^b^

SWO-TE: sunflower wax oleogel with thyme, SWO-CN: sunflower wax oleogel with cumin. ^†^ Small letters within each row (^a^) indicate significant differences among the oleogel samples for the mean ± SD values calculated from four determinations by one-way analysis of variance and Tukey’s test (*p* ≤ 0.05, *n* = 6).

**Table 2:** The thermal properties of the prepared thyme and cumin-flavored virgin olive oil-sunflower wax oleogels.

Crystallization			
	Onsetc (°C)	Peak (Tc, °C)	ΔHc (J/g)
SWO-TE	61.37 ± 0.26 ^a†^	59.54 ± 0.14 ^a^	−11.44 ± 0.2 ^a^
SWO-CN	60.55 ± 0.04 ^b^	58.72 ± 0.10 ^b^	−8.58 ± 0.4 ^b^
**Melting**			
	**Onsetm (°C)**	**Peak (Tc, °C)**	**ΔHc (J/g)**
SWO-TE	48.41 ± 0.36 ^a^	62.53 ± 0.28 ^a^	13.64 ± 0.12 ^a^
SWO-CN	53.51 ± 0.53 ^b^	62.83 ± 0.39 ^a^	12.73 ± 0.11 ^a^

SWO-TE: sunflower wax oleogel with thyme, SWO-CN: sunflower wax oleogel with cumin. ^†^ Small letters within each individual column (^a^,^b^) indicate significant differences among the oleogel samples for the mean ± SD values calculated from four determinations by one-way analysis of variance and Tukey’s test (*p* ≤ 0.05, *n* = 6).

**Table 3:** The volatile aromatics composition of thyme and cumin-flavored virgin olive oil-sunflower wax oleogels.

			SWO-TE		SWO-CN	
RT ^†^ (min)	Volatile Compound	Aroma Definition ^††^	Mean Peak Area	Peak Value (%)	Mean Peak Area	Peak Value (%)
1.377	Ethanol	Strong alcoholic, ethereal, medical	54,150 ± 1200	0.95	86,014 ±1455	1.50
1.452	1-Propen-2-ol, acetate	Ethereal, acetic, fruity, sweet berry	38,346 ± 975	0.67	49,176 ± 1005	0.85
1.537	Acetol	Pungent, sweet caramellic, ethereal	62,931 ± 1150	1.10	Nd.	Nd.
1.538	Methyl acetate	Green, ethereal, fruity, fresh, rum and whiskey-like	Nd.	Nd.	56,446 ± 3100	0.98
1.798	Acetic acid	Sharp, pungent, sour vinegar	178,460 ± 11,845	3.13	159,524 ± 8900	2.77
1.920	Ethyl acetate	Ethereal, fruity, sweet, weedy green	45,398 ± 2187	0.80	59,232 ± 1838	1.03
2.565	1-Penten-3-ol	Horseradish, tropical fruity	46,779 ± 2400	0.82	44,253 ± 2450	0.77
2.716	Diethyl ketone	Ethereal acetone	237,320 ± 3612	4.16	Nd.	Nd.
4.679	Hexanal	Fresh green, fatty, aldehydic, grass, leafy fruity, sweaty	443,159 ± 1560	7.77	339,392 ± 1365	5.90
6.174	(E)-2-Hexenal	Fresh green, leafy, fruity with rich vegetative nuances	756,741 ± 1200	13.27	512,621 ± 6845	8.91
6.645	2-Hexen-1-ol, (Z)-	Fresh vegetative, slightly fatty with a green bean note	91,771 ± 3150	1.61	Nd.	Nd.
10.339	beta-Phellandrene	Mint terpentine	Nd.	Nd.	36,041 ± 855	0.63
10.489	beta- Pinene	Herbal, terpenic	Nd.	Nd.	75,343 ± 505	1.31
10.865	6-Methyl-5-hepten-2-one	Citrus, green, musty, apple	67,412 ± 2007	1.18	Nd.	Nd.
11.020	Geranyl butyrate	Sweet fruity, rose, waxy, raspberry	79,389 ± 3860	1.39	Nd.	Nd.
11.022	beta-Myrcene	Spicy	Nd.	Nd.	90,276 ± 1286	1.57
11.610	l-Phellandrene	Minty	Nd.	Nd.	45,754 ± 575	0.80
11.708	delta-3-Carene	Citrus, terpenic, herbal, medicinal	Nd.	Nd.	77,918 ± 860	1.35
12.318	Cymol	Terpenic	99,598 ± 555	1.75	449,216 ± 1258	7.81
12.505	Limonene	Terpene, pine, herbal, peppery	1,690,329 ± 18,525	29.65	1,319,846 ± 18,555	22.94
13.220	beta-Ocimene	Citrus, tropical, green, terpene, woody	37,854 ± 3150	0.66	Nd.	Nd.
13.645	gamma-Terpinene	Terpy, citrus, lime-like, oily, green	60,257 ± 1100	1.06	803,955 ± 7500	13.98
15.494	Nonanal	Effervescent, aldehydic citrus, cucumber and melon rindy	69,958 ± 3154	1.23	26,362 ± 745	0.46
20.662	Propanal, 2-methyl-3-phenyl-	Aldehydic	Nd.	Nd.	386,598 ± 1008	6.72
22.281	2-Caren-10-Al	Citrus	Nd.	Nd.	112,619	1.96
22.785	Carvacrol	Spicy, herbal, phenolic, medicinal and woody	183,194 ± 2500	3.21	Nd.	Nd.
27.392	Bergamotene <alpha-trans->	Woody	28,178 ± 269	0.49	Nd.	Nd.
27.629	Cedrene <beta->	Cedarwood woody	45,190 ± 3180	0.79	Nd.	Nd.
29.701	Farnesene	Woody	137,168 ± 12,456	2.41	66,899 ± 3820	1.16
30.311	Sesquiphellandrene <beta->	Sweet, fruity, herbal	51,413 ± 1873	0.99	Nd.	Nd.

^†^ RT: retention time, ^††^ Aromatic definitions of the volatile compounds were found from the web page: https://www.thegoodscentscompany.com/index.html#. SWO-TE: sunflower wax oleogel with thyme, SWO-CN: sunflower wax oleogel with cumin.

**Table 4:** The sensory quantitative descriptive analysis (QDA) results of the prepared thyme and cumin-flavored virgin olive oil-sunflower wax oleogels.

	SWO-TE	SWO-CN
Hardness	8.15 ± 0.5 ^a,†^	8.25 ± 0.3 ^a^
Spreadability	10.00 ± 0.0 ^a^	10.00 ± 0.0 ^a^
Liquefaction	0.55 ± 0.0 ^a^	0.50 ± 0.0 ^a^
Sandiness	1.00 ± 0.0 ^a^	1.00 ± 0.0 ^a^
Olive fruit	4.20 ± 0.5 ^a^	4.00 ± 0.5 ^b^
Grassy	5.15 ± 0.2 ^b^	7.00 ± 0.2 ^a^
Waxy	0.50 ± 0.0 ^a^	0.50 ± 0.0 ^a^
Rancid	0.52 ± 0.3 ^a^	0.50 ± 0.3 ^a^
Thyme	5.23 ± 0.5 ^a^	0.00 ± 0.0 ^b^
Cumin	0.00 ± 0.0 ^b^	3.00 ± 0.0 ^a^
Hay	1.00 ± 0.0 ^a^	1.05 ± 0.0 ^a^
Cooling	2.05 ± 0.1 ^b^	2.55 ± 0.2 ^a^
Mouth coating	7.45 ± 0.4 ^a^	7.00 ± 0.4 ^a^

SWO-TE: sunflower wax oleogel with thyme, SWO-CN: sunflower wax oleogel with cumin. ^†^ Small letters within each row (^a^,^b^) indicate significant differences among the oleogel samples for the mean ± SD values calculated from four determinations by one-way analysis of variance and Kruskal–Wallis test (*p* ≤ 0.05, *n* = 6).

**Table 5:** The consumer test results of the prepared thyme and cumin-flavored virgin olive oil-sunflower wax oleogels.

	SWO-TE	SWO-CN
Appearance	4.30 ± 0.7 ^a,†^	4.04 ± 0.8 ^a^
Spreadability	4.40 ± 0.7 ^a^	4.14 ± 0.8 ^a^
Aroma	4.18 ± 0.8 ^a^	4.18 ± 0.8 ^a^
Flavor	4.36 ± 0.8 ^a^	4.24 ± 0.8 ^a^
Acceptability	4.26 ± 0.7 ^a^	4.16 ± 0.7 ^a^

SWO-TE: sunflower wax oleogel with thyme, SWO-CN: sunflower wax oleogel with cumin. ^†^ Small letters within each row (^a^) indicate significant differences among the oleogel samples for the mean ± SD values calculated from four determinations by one-way analysis of variance and Kruskal–Wallis test (*p* ≤ 0.05, *n* = 6).

**Table 6:** The panel defined sensory descriptive terms, their definitions, and references used.

	Definition	References
Hardness	Force required to push a knife into the sample	Min: Yoghurt, Max: Tallow
Spreadability	Easiness of deploying sample over a bread loaf	Min: Chewing gum, Max: Cream cheese
Liquefaction	Amount of fat melting after the sample was spread on bread surface	Min: Tallow, Max: Olive oil
Sandiness	The perceived gritty texture on tongue	Min: Absent, Max: Semolina
Olive fruit	The flavor and aroma of fresh green olives	Min: Absent, Max: Green olive
Grassy	The aroma of fresh cut grasses	Min: Absent, Max: Cut grass
Waxy	Aromas associated with waxes	Min: Absent, Max: Paraffin wax
Rancid	Aromas associated with oxidized oil	Min: Absent, Max: Used frying oil
Thyme	Aromas associated with thyme spice	Min: Absent, Max: Ground thyme
Cumin	Aromas associated with cumin spice	Min: Absent, Max: Ground cumin
Hay	The aromatics associated with sweet, dry grasses	Min: Absent, Max: Dry hay
Cooling	Cold feeling inside mouth	Min: Absent, Max: Menthol candy
Mouth coating	The perceived fatty coating inside mouth space	Min: liquid oil, Max: Butter

## Data Availability

Data is contained within the article.
